# Development of a Laser Microdissection-Coupled Quantitative Shotgun Lipidomic Method to Uncover Spatial Heterogeneity

**DOI:** 10.3390/cells12030428

**Published:** 2023-01-28

**Authors:** Vanda Varga-Zsíros, Ede Migh, Annamária Marton, Zoltán Kóta, Csaba Vizler, László Tiszlavicz, Péter Horváth, Zsolt Török, László Vígh, Gábor Balogh, Mária Péter

**Affiliations:** 1 Institute of Biochemistry, Biological Research Centre, Eötvös Loránd Research Network, 6726 Szeged, Hungary; 2 Faculty of Science and Informatics, Ph.D. School in Biology, University of Szeged, 6726 Szeged, Hungary; 3 Single Cell Omics Advanced Core Facility, Hungarian Centre of Excellence for Molecular Medicine, 6726 Szeged, Hungary; 4 Albert Szent-Györgyi Medical Centre, Department of Pathology, University of Szeged, 6725 Szeged, Hungary

**Keywords:** cryosection, laser microdissection, in situ lipid extraction, mass spectrometry, quantitative shotgun lipidomics, spatial resolution, tissue heterogeneity

## Abstract

Lipid metabolic disturbances are associated with several diseases, such as type 2 diabetes or malignancy. In the last two decades, high-performance mass spectrometry-based lipidomics has emerged as a valuable tool in various fields of biology. However, the evaluation of macroscopic tissue homogenates leaves often undiscovered the differences arising from micron-scale heterogeneity. Therefore, in this work, we developed a novel laser microdissection-coupled shotgun lipidomic platform, which combines quantitative and broad-range lipidome analysis with reasonable spatial resolution. The multistep approach involves the preparation of successive cryosections from tissue samples, cross-referencing of native and stained images, laser microdissection of regions of interest, in situ lipid extraction, and quantitative shotgun lipidomics. We used mouse liver and kidney as well as a 2D cell culture model to validate the novel workflow in terms of extraction efficiency, reproducibility, and linearity of quantification. We established that the limit of dissectible sample area corresponds to about ten cells while maintaining good lipidome coverage. We demonstrate the performance of the method in recognizing tissue heterogeneity on the example of a mouse hippocampus. By providing topological mapping of lipid metabolism, the novel platform might help to uncover region-specific lipidomic alterations in complex samples, including tumors.

## 1. Introduction

Lipids are crucial players in a variety of biological processes [1], for example, they are key factors in stress sensing and signaling [2,3]. Lipid changes induce alterations in the propagation of cell messages that can be associated with pathological states, such as cardiovascular and neurodegenerative diseases, diabetes, or cancer [4,5,6,7]. There is therefore a continuous effort to develop a reliable, high-throughput, sensitive, and automatic method for the global analysis of lipids at the cellular level [8]. The most suitable method to achieve this goal is high-resolution mass spectrometry (MS) [9], including shotgun lipidomics, which provides a quantitative snapshot of the lipidome within a short period of time without chromatographic separation [10,11].

Tissues and tumors are composed of multiple distinct regions, and these regions also consist of multiple cell types possessing distinct characteristics. In parallel with the structural and compositional characterization, it is equally important to elucidate the spatial distribution of lipids, which can provide a hitherto unexplored, novel molecular base for drug discovery and disease treatment [12]. Mass spectrometry imaging (MSI) techniques, such as matrix-assisted laser desorption ionization (MALDI) [13,14] or desorption electrospray ionization (DESI) [15], achieve spatial resolution at the low micrometer range, and have already provided important insights into the localization and abundance of small metabolites and lipids in tissue sections. However, signal variability in MSI, caused by matrix effects and extraction efficiency, may be detrimental to accurate quantification [16]. It follows that compensation for these effects is necessary and requires different normalization strategies and/or addition of matrix additives and appropriate internal standards to facilitate quantitative MSI [17].

An alternative approach is to use laser (capture) microdissection (LMD) that employs a laser to ablate and catapult the material typically from a 5–20 µm thick cryosection. The catapulted material can be collected, processed further, and subjected to different omics analysis. LMD-coupled RNA sequencing [18,19,20,21] and proteomics [22,23,24,25] allowed the better understanding of the cell type-specific landscape and aided the analysis of spatial organization. In lipidomics (and metabolomics), the acquisition of both in-depth spatial information and comprehensive lipidome coverage is extremely difficult, especially when biological material is limited or lipids are at low abundance [26]. In one of the few examples, Knittelfelder et al. demonstrated the quantification of hundreds of lipids isolated by LMD from liver histological zones covering 0.3–0.5 mm^2^ area [27]. Similarly, the lipid profiles of cell body- and synapse-enriched regions of the Drosophila brain, equivalent to a sample amount of 50 cells, were determined and found to be distinct by using LMD, fluorescence microscopy, and liquid chromatography-multistage MS [28].

Here we present the development and validation of a novel LMD-coupled shotgun lipidomic platform, which enables rapid, simple, quantitative, and broad-range lipidome analysis with spatial resolution down to 80 µm. We show that the developed protocol has the capacity to provide valuable topological information in a wide variety of applications.

## 2. Materials and Methods

### 2.1. LMD-Coupled Shotgun Lipidomic Platform Overview

The LMD-coupled shotgun lipidomic platform that we developed in this work includes the following steps: preparation of successive native and hematoxylin-eosin (HE)-stained cryosections from tissue samples, high-resolution microscopy followed by cross-referencing of native (autofluorescence) and HE-stained images, marking of region of interests (ROIs) in the native image, automated LMD into 96-well plates, in situ lipid extraction, direct sample delivery by robotic nanoflow injection, and quantitative, high-resolution shotgun lipidomics.

### 2.2. Samples

#### 2.2.1. Mouse Tissue Samples

C57BL/6 wild-type mice, used in this study, were handled in accordance with the standards established by the EU Directive 2010/63/EU, and the experiments were approved by the regional Station for Animal Health and Food Control (Csongrád County, Hungary; project license: XVI/766/2018). Following cervical dislocation, the liver, kidney, and brain were removed, washed with PBS (pH = 7.4), embedded in cryomolds with O.C.T (optimal cutting temperature) mounting medium (VWR, Leuven, Belgium), frozen on dry ice, and stored at −80 °C until further processing.

#### 2.2.2. HeLa-Kyoto 2D Cell Culture

HeLa-Kyoto human adenocarcinoma cells (CLS Cell Lines Service GmbH, Eppelheim, Germany), stably expressing two fluorescent proteins, a H2B-associated mCherry fluorescent protein and an EGFP-alpha tubulin-associated GFP, were cultured in selection medium (DMEM with 4.5 mg/mL glucose, supplemented with 10% FBS, 2 mM glutamine, 0.5 mg/mL geneticin (G418) and 0.5 μg/mL puromycin) at 37 °C. For LMD, the cells were seeded on PET membrane frame slides (Leica Microsystems, Wetzlar, Germany) at 50,000 cells/cm^2^ density. Prior to seeding, the membrane was washed with 70% ethanol for 30 min, distilled water, and PBS, and finally exposed to UV light for 30 min. After 1 day incubation at 37 °C, the cells reached about 80% confluency, and could be subjected to LMD, as described in Section 2.6.

### 2.3. Cryosectioning

The frozen, O.C.T.-embedded tissue piece was placed into the chamber of a Leica CM1860 UV cryostat (Leica Biosystems, Deer Park, IL, USA) and allowed to equilibrate for 20 min at −18 °C. Successively, 10 µm thick cryosections were obtained at −18 °C (blade/chamber temperature). Depending on further processing, the resulting cryosections were thaw-mounted on microscopic or membrane-coated microscopic glass slides.

### 2.4. Hematoxylin-Eosin Staining

For hematoxylin-eosin (HE) staining, the tissue cryosections were mounted on a microscopic glass slide (Superfrost Microscope Slide, VWR, Leuven, Belgium), equilibrated at room temperature, fixed in 10% formalin solution (Sigma-Aldrich, St. Louis, MO, USA) for 30 min, washed with distilled water and dried at room temperature. For nuclei staining, the slides were placed in hematoxylin (Sigma-Aldrich, St. Louis, MO, USA) solution for 5 min, then washed with running tap water for 4 min and counterstain with eosin (Sigma-Aldrich, St. Louis, MO, USA) for better visibility of the cytoplasm. Following the eosin staining, the slides were washed with distilled water, dehydrated with increasing concentrations of alcohol, and finally covered with a xylene-based mounting medium (Shandon Consul-Mount, Thermo Scientific, Runcorn, United Kingdom).

### 2.5. High-Resolution Microscopy and Marking Regions of Interest (ROIs)

Successive, native (autofluorescence), and HE-stained sections were screened using a Panoramic 250 Flash III scanner (SYSMEX EUROPE SE, Norderstedt, Germany). High-resolution whole-slide images were obtained by 20×/0.80 and 40×/0.95 objectives. An sCMOS (pco.edge 4.2 bi) 8-bit camera was applied to obtain fluorescent images (DAPI, FITC, TRTTC, Cy5, Cy7), and an Adimec QUARTZ Q-12 A180 camera for brightfield images. Lumencor SPECTRA III L illumination was used during image acquisition.

High-resolution brightfield and/or autofluorescence images of the native sections were matched to the images of the HE-stained sections by cross-referencing to specific locations, and ROIs were marked in the native images using the Biology Image Analysis Software ((BIAS) Single Cell Technologies, Szeged, Hungary) [25].

### 2.6. Automated Laser Microdissection (LMD)

For laser microdissection (LMD), tissue cryosections were mounted onto a microscopic glass slide covered with polyethylene naphthalate (PEN) membrane with a thickness of 2 µm (Leica Microsystems, Wetzlar, Germany) which was previously exposed to UV light at 254 nm for 30 min. Slides were stored at −80 °C for maximum 2–3 days before LMD and subsequent MS analysis. As described above ( 2.2.2), cell culture samples were directly seeded on membrane frame slides.

To appropriately excise different sample areas, we used the Leica LMD6000 laser microdissection microscope (Leica Microsystems, Wetzlar, Germany) with an adjustable and flexible diode-pumped solid-state laser (355 nm, maximum pulse energy: 70 μJ). High cutting precision was achieved using HC PL FLUOTAR L 20×/0.40 CORR, 40×/0.60 XT, or 63×/0.70 CORR XT objectives and Leica DFC7000 T CCD camera. The LMD system was controlled by the Leica Laser Microdissection V 8.2.3.7603 software. Areas from 5000 to 160,000 µm^2^ were excised, and each dissected sample piece was collected separately in a single well of a 150 µL 96-well plate (Eppendorf, Hamburg, Germany). The LMD spots were processed further without sample transfer as described in Section 2.7.

### 2.7. In Situ Lipid Extraction

Single-step, one-phase lipid extraction was performed directly in the wells of a 150 µL 96-well plate which were used previously to capture the LMD samples. We developed a chloroform:methanol:isopropanol mixture (1:2:1, by vol.) as an extraction solvent which contained 0.3% dimethylformamide (DMF) and 0.3 mM ammonium chloride (NH_4_Cl) as well as a set of MS quantification standards (Table S1). This mixture was then directly used as an infusion solvent for shotgun MS measurements. The concentration of the extraction/infusion solvent was adjusted between 0.02 and 0.10 µg wet weight (ww)/solvent µL. The well plates were first centrifuged and then the extraction solvent was pipetted in. After sealing, the plates were shaken for 5 min and left to stand for 1 h (at 8 °C) before the MS measurements.

### 2.8. Shotgun Mass Spectrometry

Lipidomic standards were from Avanti Polar Lipids (Alabaster, AL, USA). Solvents for extraction and MS analyses were Optima LC-MS grade (Thermo Fisher Scientific, Waltham, MA, USA) and liquid chromatographic grade (Merck, Darmstadt, Germany). All other chemicals were the best available grade purchased from Sigma (Steinheim, Germany) or Merck (Darmstadt, Germany).

MS analyses were performed on an Orbitrap Fusion Lumos instrument (Thermo Fisher Scientific, Bremen, Germany) equipped with a robotic nanoflow ion source (TriVersa NanoMate, Advion BioSciences, Ithaca, NY, USA) using chips with a spraying nozzle diameter of 5.5 µm. The back pressure was set at 1 psi. The ionization voltages were +1.3 kV and −1.9 kV in positive and negative modes, respectively, whereas it was +1.5 kV in acquisitions with polarity switching. The temperature of the ion transfer capillary was 260 °C. Acquisitions were performed at mass resolution R_m/z 200_ = 240,000 in full scan mode. In the polarity switching method, spectra were acquired within the mass range of *m/z* 400–1300 from 0.2 to 0.6 min in the negative and from 0.8 to 1.2 min in the positive polarity mode. Phosphatidylcholine (diacyl, PC and alkyl-acyl, PC-O), phosphatidylethanolamine (diacyl, PE and alkenyl-acyl, PE-P), phosphatidylinositol (PI), phosphatidylserine (PS), phosphatidic acid (PA), phosphatidylglycerol (PG)/bis(monoacylglycero)phosphate (BMP), cardiolipin (CL), and the lyso derivatives LPC, LPE, LPI, LPS, LPG, and LCL as well as ceramide (Cer), hexosyl ceramide (HexCer), GM3 ganglioside, and sulfatide (Sulf) were detected and quantified using the negative ion mode, whereas sphingomyelin (SM), diacylglycerol (DG), triacylglycerol (TG), and cholesteryl ester (CE) were detected and quantified using the positive ion mode. In addition, PCs were also analyzed in the positive ion mode, and we detected and profiled higher brain gangliosides (GD3, GD1, GT1, and GQ1) in the negative polarity mode.

### 2.9. Mass Spectrometry Data Analysis

Lipid species were identified by LipidXplorer 1.2.8.1 software [29]. Identification was executed by matching the *m/z* values of their monoisotopic peaks to the corresponding elemental composition constraints. Mass tolerance was set to 2 ppm. Signal intensities were integrated after built-in C13 isotopic corrections. These data files were subjected to LipidXplorer queries for annotation. Lipid species were annotated according to the classification systems for lipids [30,31] at the level of sum formulas. For glycerolipids, e.g., PC(38:6), the total numbers of carbons followed by double bonds for all chains are indicated. For sphingolipids, the sum formula, e.g., SM(36:1:2), specifies first the total number of carbons in the long chain base and fatty acid moiety then the sum of double bonds in the long chain base and the fatty acid moiety followed by the sum of hydroxyl groups in the long chain base and the fatty acid moiety. We note that the sum formula of glycero(phospho)lipids might describe different fatty acyl combination isomers. In addition, PC/PE-O(x:y) (alkyl-acyl) and PC/PE-P(x:y-1) (alkenyl-acyl) species as well as PG and BMP species are also isomeric. Because we did not perform fragmentation experiments in the current work, these types of coexisting isomers could not be resolved and were quantified collectively. Nevertheless, in Figure S3B, we demonstrate that the platform is suitable for conducting such experiments, and therefore has the capability to resolve isomeric lipid species.

Data were further processed by in-house Excel macros including intensity data filtering, sample grouping, quantification, basic statistics, and visualization. Quantification was made by comparing integrated signal intensities with those of the internal standards; the list of quantification standards and ion formats are provided in Table S1. Lipidomic data were expressed as mol% of membrane lipids, where membrane lipids were calculated as the sum of glycerophospho- and sphingolipids, or as absolute quantities expressed as lipid pmol values. Precision was described by the coefficient of variation (CV%), which was calculated as SD/Mean × 100. Linearity was validated by the regression coefficient (R^2^). Multivariate statistical analysis of brain lipidomic dataset was performed using MetaboAnalyst [32].

## 3. Results and Discussion

### 3.1. Method Overview

**Figure 1:**
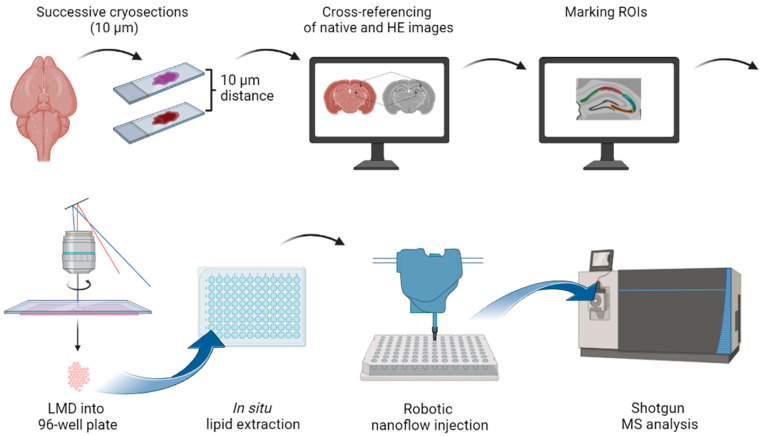
Laser microdissection (LMD)-coupled quantitative shotgun lipidomic workflow (created with BioRender.com). The workflow includes the preparation of successive native and HE-stained cryosections from tissue samples, high-resolution microscopy followed by cross-referencing of native and stained images, marking of ROIs in the native image, automated LMD into 96-well plates, in situ lipid extraction, direct sample delivery by robotic nanoflow injection, and quantitative shotgun lipidomics.

In this work we developed and validated a multistep workflow which offers lipidomic analysis with reasonable spatial resolution. The novel method includes the (1) preparation of successive native and stained cryosections from tissue samples, (2) high-resolution microscopy followed by cross-referencing of native and stained images, (3) marking of region of interests (ROIs) in the native image, (4) automated LMD into 96-well plates, (5) in situ lipid extraction, (6) direct sample delivery by robotic nanoflow injection, and (7) quantitative, high-resolution shotgun lipidomics (Figure 1).

### 3.2. Method Validation—Reproducibility and Extraction Efficiency

In the current protocol, the tissue cryosection was mounted onto a glass slide coated with PEN membrane and the entire ROI was excised by a nitrogen laser along its premarked border. As a result of ablation, a single membrane-attached piece of tissue catapulted downwards and was collected in a well of a 96-well plate. We performed one-phase lipid extraction directly in the well plate by adding a chloroform:methanol:isopropanol mixture (1:2:1, by vol.) to the LMD tissue piece. The extraction solvent contained additives to promote ionization during the subsequent MS process as well as a set of MS quantification standards.

**Figure 2:**
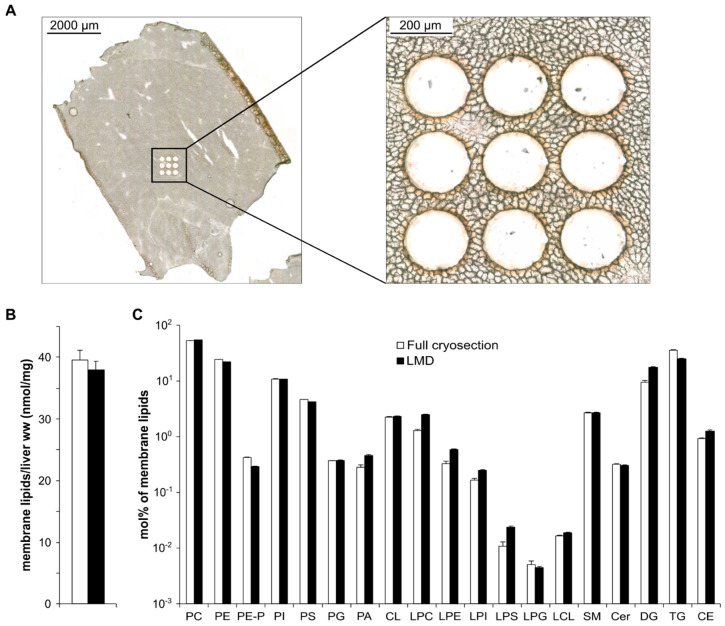
Validation of the LMD-coupled shotgun lipidomics. (**A**) Locations of ROIs after LMD in a representative brightfield image showing the full mouse liver cryosection (**left**) and a zoom-in view (**right**). (**B**) Extracted membrane lipid amount expressed as nmol/mg liver wet weight (ww). (**C**) Lipid class composition of dissected ROIs expressed as mol% of membrane lipids. Data represent mean ± SEM (n = 4 for full cryosections used as controls; n = 24 for LMD spots, 6−9 spots from 3 successive sections).

First, we showed that the in situ one-phase lipid extraction provided efficient and reproducible lipid extraction from the LMD spots. We compared the extraction efficiency in terms of both quantitativity and lipid composition with our previously validated one-phase methanolic extraction [33]. Successive, close–distant (10 µm distance), 10 µm thick cryosections were prepared from a small piece of a mouse liver (7 mm × 7 mm; ca. 490 µg ww) (Figure 2A, left), and either extracted directly in 250 µL methanol (4 parallel sections) or subjected to LMD. In the latter case, circles of 30,000 µm^2^ in size were dissected (altogether 24 individual spots from 3 parallel sections, ca. 0.3 µg ww each), as indicated in Figure 2A (right). The close vertical and horizontal distance of the LMD spots ensured that the catapulted areas represented homogeneous tissue regions. The spots were in situ extracted and, without sample transfer, the extracts were directly infused into the mass spectrometer. The full cryosection-derived methanolic extracts were diluted further before the MS measurement so that the concentrations of the MS infusion solvents were comparable in the laser-ablated and non-laser-ablated sample types (0.06–0.10 µg ww/infusion solvent µL).

We identified and quantified ca. 200 lipid species (at sum formula level) including structural, signaling, and storage lipids. We set the upper limit of coefficient of variation (CV%) to 30. This criterion left 164 eligible components, 66% of which displayed good to excellent CV% values (≤20), and thereby verified the reproducibility of the workflow (Table S2).

To assess the extraction efficiency, we used the non-laser-ablated, full cryosections as controls. For membrane lipids, such as glycerophospholipids and sphingolipids, we found that both the recovered lipid amount and the extracted lipid profile of LMD samples were in good agreement with those of the full cryosections (Figure 2B,C and Table S2). We also note that the lipid content matched well with previously published data for mouse liver [34,35]. For neutral lipids DG, TG, and CE, the observed differences between data originating from the full slices versus the LMD spots might indicate that the total area of LMD samples represented only a small fraction of the full cryosection, which can be more heterogeneous for these lipids. In addition, we note the consistently larger relative amount of lysolipids and PA in the LMD samples compared with the non-dissected controls (3.8 vs. 2.1 mol%). This might reflect slight degradation of structural lipids caused by laser ablation, but the difference in their sum amounts did not refer to severe decomposition, in contrast, it proved the reliability of the method.

### 3.3. Method Validation—MS Acquisition with Polarity Switching

**Figure 3:**
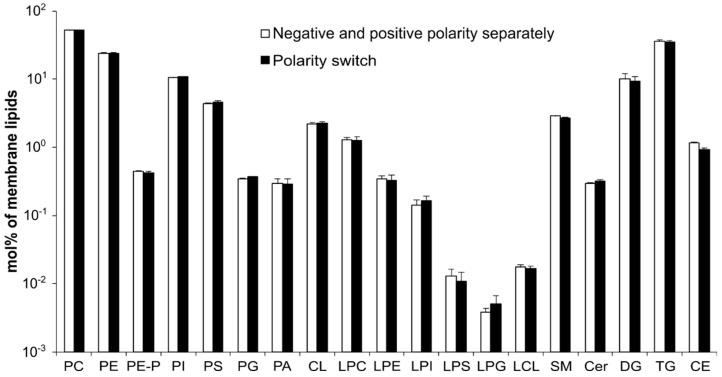
Comparison of lipidomic profiles from MS measurements conducted separately in the negative and positive ion modes with those obtained with polarity switching. Data represent mean ± SD (n = 4, methanolic extracts from full cryosections).

To maximize lipidome coverage, in our previous studies we conducted MS measurements in both polarity modes. This required the dilution of the (methanolic) extract with an appropriate amount of infusion solvent mixture, division of the diluted extract into halves, and spiking each halves with ion mode-specific additives [36,37,38]). However, when we tried to down-scale this protocol to only a few µL extraction/infusion solvent, the multiple sample transfer resulted in significant deterioration of the signal-to-noise ratio. To overcome this problem, we took advantage of high-resolution mass spectrometers which enable rapid polarity switching with sub-ppm mass accuracy, and thereby simplify and accelerate shotgun lipidomics analyses [27,39]. In the current work, supplementation of the chloroform:methanol:isopropanol mixture (1:2:1, by vol.) with dimethylformamide and ammonium chloride at low concentrations (0.3% and 0.3 mM, respectively) ensured good-quality MS spectra both in the negative and positive polarity modes from a single run within 2 min (Figure S1). Because this mixture can be used both as an extraction and infusion solvent for shotgun lipidomics, we saved time and effort by sparing the usual steps of extraction solvent evaporation, reconstitution of the extract in an appropriate infusion solvent, and double sample injection due to polarity-dependent solvent composition. The comparison of MS acquisitions conducted separately in the negative and positive polarity modes with those obtained with polarity switching provided well-matching lipidomic profiles as tested with the full cryosection methanolic extracts (Figure 3).

Nevertheless, we would like to add some remarks to mass spectra acquisition with polarity switching. Phosphatidylcholine (PC), the most abundant mammalian membrane phospholipid, is often quantified from the positive polarity mode. As assessed in our previous works, ammonium chloride at mM concentrations (3–10 mM) efficiently suppressed the formation of sodiated adducts of PC compared with its protonated form (~1%). Lowering the additive amount to 0.3 mM, as in the present protocol, led to the sizeable presence of [PC+Na]+ (accounting for ca. 10% of the protonated adduct). This raised the problem of isobaric overlap between the sodiated ion of a given PC species and the protonated ion of the PC species with two additional CH_2_ and three double bonds, e.g., between [PC(34:2)+Na]+ and [PC(36:5)+H]+ (Δ*m/z* = 0.0024). Indeed, sodium adduct formation introduces the major isobaric interferences observed for phospholipids even at high mass resolution, and might cause overestimation of certain polyenoic species. Although the extent of this kind of shift depends on the species profile of PC in the given sample, it needs to be controlled and corrected. Such a possibility is offered exactly by the presence of ammonium chloride, which promotes efficiently the formation of [PC+Cl]− ions in the negative polarity mode. This allows the comparison of the species profiles for the two adduct forms, i.e., the protonated vs. chloride adduct ions of PC, which can help to avoid misquantification (Figure S2). Another alternative is a mathematically precise solution, which was introduced by Höring et al. who presented an algorithm to correct for this overlap [40]. The problem is often overlooked, especially in MSI experiments, and leads not only to lipid species overestimation but also to misidentification. On the other hand, the lower NH_4_Cl concentration significantly decreases the ammonium adduct formation of neutral lipids (DG, TG and CE). Consequently, their detection sensitivity decreases and therefore the neutral lipid content can be reliably measured by this protocol only when it is sizeable in a sample (e.g., in the liver, muscle, or adipose tissue).

### 3.4. Method Validation—Linearity of Quantification

#### 3.4.1. Kidney Cryosections

**Figure 4:**
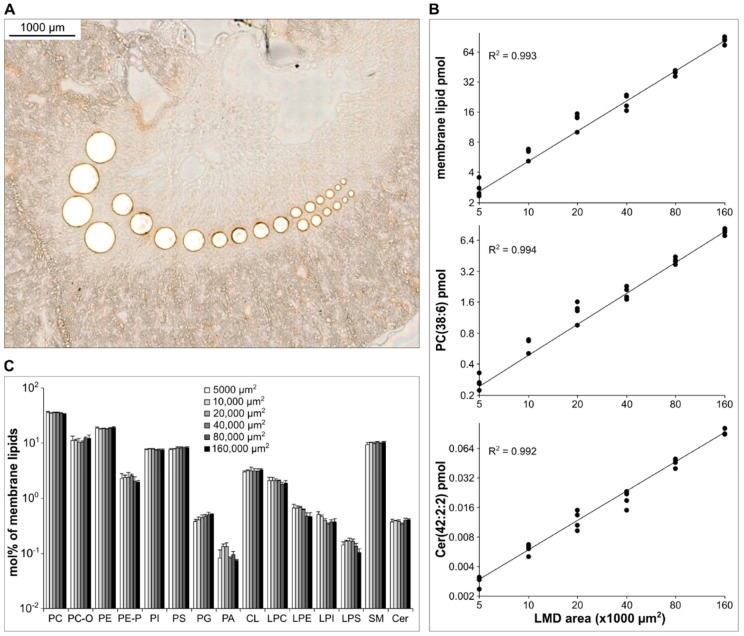
Linearity testing of the LMD-coupled shotgun lipidomics on the example of a kidney cryosection. (**A**) Representative brightfield image with locations of ROIs after LMD. (**B**) Linearity of quantification for the total membrane lipid content (**top**), for an abundant membrane lipid species, PC(38:6) (**middle**), and for a low-amount lipidome component, Cer(42:2:2) (**bottom**); (n = 4). (**C**) Membrane lipidome composition in the examined LMD range (data are expressed as mol% of membrane lipids and represent mean ± SD, n = 4, successive sections).

Next, we dissected tissue pieces from a homogeneous medulla section of a 10 µm thick kidney cryosection to determine the range of linearity of quantification. The LMD pieces, from 5000 to 160,000 µm^2^ area in size (Figure 4A), were in situ extracted; the volume of the extraction solvent was adjusted to the catapulted tissue weight (0.02–0.08 µg/solvent µL). The total membrane lipid content displayed excellent linearity over the investigated LMD range (R^2^ = 0.993, Figure 4B, top). Moreover, we observed high R^2^ values (>0.9) for all eligible components of the membrane lipidome (Table S3; eligibility criterion was R^2^ > 0.7), as exemplified by the abundant membrane lipid species PC(38:6) (Figure 4B, middle) or by the low-amount signaling lipid Cer(42:2:2) (Figure 4B, bottom). The number of quantifiable membrane lipid species was ca. 130 components at and above 10,000 µm^2^ LMD area from a 10 µm thick section (Table S3), which represented a volume of 100,000 µm^3^ and corresponded to about 25 cells (calculated with a typical mammalian cell volume of 4000 µm^3^). We note that this number dropped to only 115 components at the LMD size of 5000 µm^2^. Importantly, the observed lipidome-wise linearity of quantification was paralleled by a well-retained lipidome composition throughout the dissected area range (Figure 4C), and most of the quantified species displayed CV% < 30 values (Table S4).

#### 3.4.2. HeLa 2D Cultures

**Figure 5:**
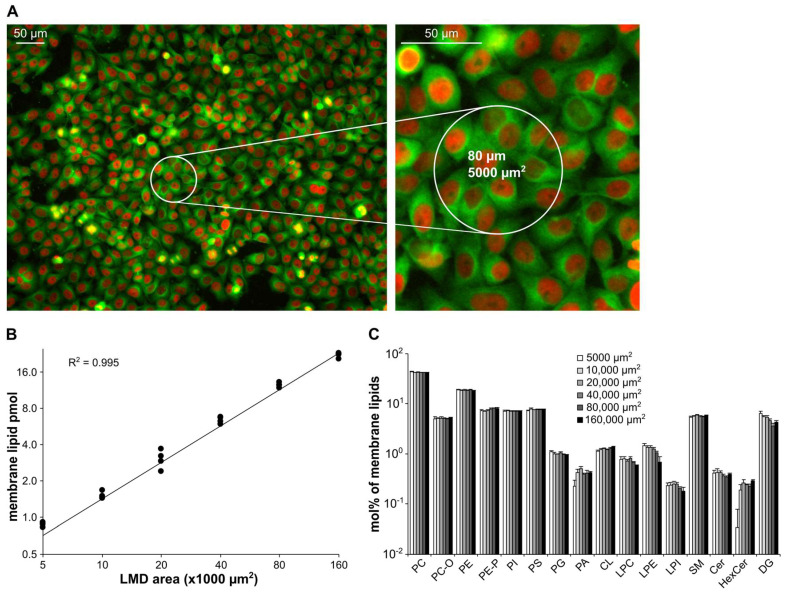
Linearity testing of the LMD-coupled shotgun lipidomics on the example of a HeLa 2D cell culture. (**A**) Representative image of the HeLa-Kyoto human adenocarcinoma cell line stably expressing two fluorescent proteins cultured to 80% confluency. (**B**) Linearity of quantification for the total membrane lipid content (n = 4). (**C**) Membrane lipidome composition over the dissected LMD range (data are expressed as mol% of membrane lipids and represent mean ± SD, n = 4).

We also tested the novel LMD-coupled shotgun lipidomics protocol on a 2D mammalian cell culture model. HeLa cells were grown to 80% confluency (Figure 5A) and areas from 5000 to 160,000 µm^2^ in size were dissected. Similarly to the tissue example, the total membrane lipid content showed excellent linearity (R^2^ = 0.995, Figure 5B) with most of the individual lipid species displaying high R^2^ values (R^2^ > 0.9, Table S5). We quantified ca. 140 components from LMD areas at and above 20,000 µm^2^ (Table S5). This number dropped to 120 at LMD size of 10,000 µm^2^. We could quantify 95 lipid species from the 5000 µm^2^ LMD spot, which corresponded to about 10 cells and represented less than 1 pmol total membrane lipid content (Figure 5A,B). In parallel, the lipidome composition proved to be well-maintained throughout the investigated LMD range (Figure 5C). For most of the quantified species, we found CV% < 30 values even for the lowest LMD size (Table S6).

### 3.5. Recognition of Hippocampus Heterogeneity

**Figure 6:**
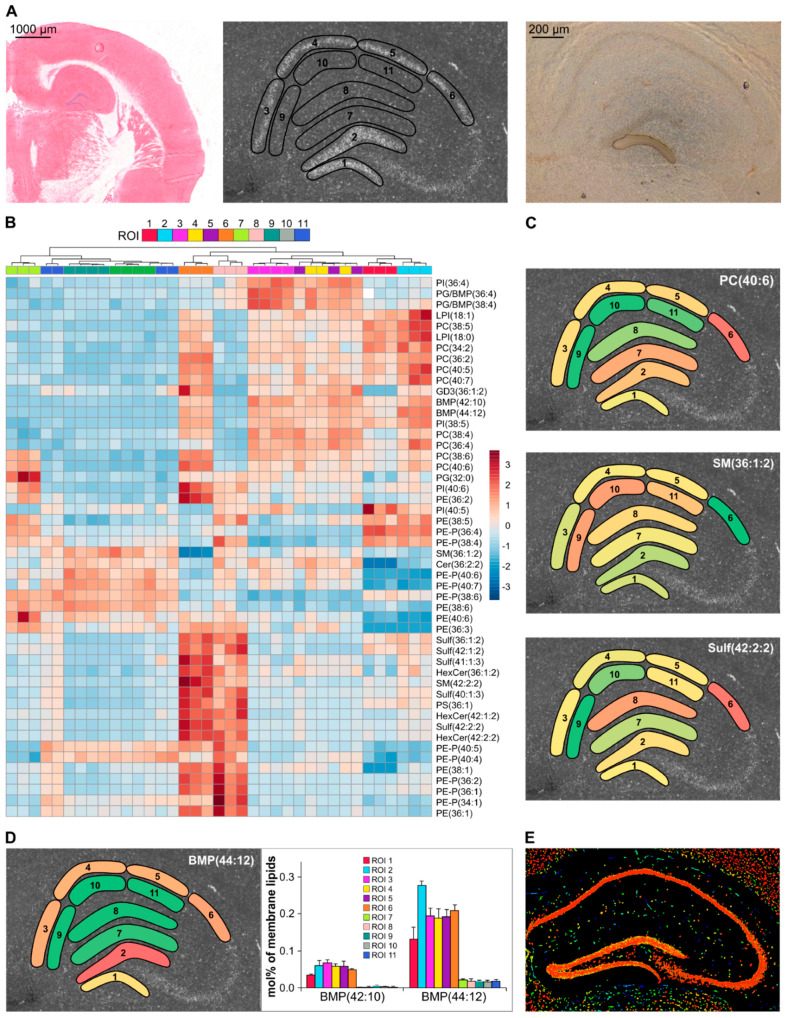
Spatial heterogeneity of the mouse hippocampus lipidome. (**A**) Representative image of a HE-stained hemibrain cryosection (**left**), autofluorescence image with the location of ROIs to be dissected (**center**), and LMD in progress (**right**). (**B**) Heatmap representation of hierarchical cluster analysis; distance measure, Euclidean; clustering algorithm, Ward; heat color code represents normalized values (z-scores). (**C**) Spatial distribution of selected lipid species, PC(40:6), SM(36:1:2), and Sulf(42:2:2). (**D**) Spatial distribution of BMP(44:12) (**left**) and quantitative assessment of BMP(44:12) and BMP(42:10) (**right**; data represent mean ± SD, n = 3–4, successive sections). (**E**) Reference image for Cathepsin B expression taken from the Allen Brain Institute website atlas.brain-map.org. ROI 1 and 2, granule cell layers of the dentate gyrus; ROI 3–5, stratum pyramidale layer of the CA1 region; ROI 6, stratum pyramidale layer of the CA2 region; ROI 7, molecular cell layer of the dentate gyrus; ROI 8, stratum lacunosum-moleculare layer of the CA1 region; and ROI 9–11, stratum radiatum layer of the CA1 region. PC: phosphatidylcholine; SM: sphingomyelin; Sulf: sulfatide; BMP: bis(monoacylglycero)phosphate.

To test the performance of the developed platform in recognizing tissue heterogeneity, the hippocampus region of 10 µm thick mouse brain cryosections were subjected to LMD; altogether 11 distinct areas were dissected (between ca. 50,000 and 130,000 µm^2^, Figure 6A) from 3 to 4 successive sections, including areas of the granule cell (ROIs 1 and 2) and molecular (ROI 7) layers of the dentate gyrus (DG) as well as the stratum pyramidale (ROI 3–6), the stratum lacunosum-moleculare (ROI 8), and the stratum radiatum (ROI 9–11) layers of the different Cornu Ammonis (CA) regions. As shown in the cluster heatmap in Figure 6B, MS analysis revealed that the anatomically similar structures showed similar lipidomic patterns (e.g., granule cell layer of DG (ROIs 1 and 2), pyramidal cell layer of CA1 (ROIs 3–5) or stratum radiatum layer of CA1 (ROIs 9–11)), whereas those that are known to be anatomically distinct displayed significantly different lipidomic fingerprints (Figure 6B). Interestingly, we observed very specific lipidome composition for the stratum pyramidale layer in the CA2 (ROI 6) versus CA1 (ROIs 3–5) regions. This observation is in line with the rediscovery of the CA2 subfield revealing its unique properties and functions [41].

Examples for the spatial distribution of selected lipid species are depicted in Figure 6C (the whole hippocampal dataset is provided in Table S7). We chose these molecules to represent different lipid classes from the brain lipidome, such as the glycerophospholipid PC(40:6), the phosphosphingolipid SM(36:1:2), and the characteristic brain acidic glycosphingolipid Sulf(42:2:2). The spatial distribution of PC(40:6) and Sulf(42:2:2) displayed significant enrichment in the stratum pyramidale layer of the CA2 region (ROI 6), whereas SM(36:1:2) appeared in lower abundance in this area. Remarkably, the distribution of several hippocampal lipid species, such as PC(40:6) or SM(36:1:2), matched well with recently published results obtained with an MSI technique [42]. In addition, the left panel in Figure 6D demonstrates the spatial distribution of BMP(44:12) with its enrichment in the neuronal layers (ROIs 1, 2, and 3–6), whereas the right panel represents a bar chart to better illustrate the quantitative assessment of this species together with BMP(42:10). Although we cannot dissect the isomeric BMP (bis(monoacylglycero)phosphate) and PG (phosphatidylglycerol), the highly unsaturated species composition is known to be characteristic for the lysosomal marker lipid BMP [43]. Notably, the observed topology for these lysosomal lipid species correlates well with the hippocampal distribution pattern of Cathepsin B (Figure 6E, taken from the Allen Mouse Brain Atlas [44]), a powerful lysosomal protease, which was recently reported as a rational drug target for a wide range of neurological disorders [45]. Finally, we note that the successive cryosections revealed excellent reproducibility for all ROIs, as shown in the cluster heatmap in Figure 6B or indicated in the right panel of Figure 6D even for species that account for less than 0.3 mol% of the membrane lipidome.

### 3.6. Method Limitations and Potentials

MSI techniques are capable of high spatial resolution but often do not provide quantitative data and possess limited lipidome coverage [16,17,46]. To date, there are only a few publications that report quantitative measurements with reasonable spatial resolution. Knittelfelder et al. developed a powerful LMD-lipidomic platform that can provide quantitative data with broad lipidome coverage (ca. 200 lipid species) obtained from LMD areas of 300,000–500,000 µm^2^ in size from a 20 µm thick liver cryosection, thereby enabling the differentiation of periportal and pericentral histological zones [27]. The authors attempted to recover lipids directly in isopropanol which filled the collection cap in their LMD setup. However, such elimination of a separate extraction step failed because they could recover much lower lipid amount compared with a well-established extraction method. In comparison, the workflow presented in the current work follows similar principles but, because we could develop a suitable solvent/additive system, it is simpler regarding the extraction and infusion steps and provides remarkably higher spatial resolution (down to 5000 µm^2^) while retaining quantitativity, linearity, and coverage.

The absence of chromatographic separation sets the major limitation of the shotgun strategy because of the ion suppression caused by the “all in one” sample delivery. In addition, it also prevents the resolution of various isomeric and isobaric molecules. Nevertheless, a major advantage of the approach is that MS spectra can be recorded under a constant concentration of the infusion solution without time constraints [10]. Indeed, the infusion of a few µL sample with a nanoflow injection system, as applied in the current protocol, enables mass spectra acquisition for minutes (Figure S3A). This allows the execution of “unlimited” number of fragmentation experiments (MS2 and MS3) to resolve fatty acyl composition of glycerophospholipids (Figure S3B). In addition, the application of different selected ion monitoring or multiple reaction monitoring methods, or even the performance of spectrum stitching, is possible to improve detection sensitivity. The latter technique was effectively applied in the shotgun lipidomic platform developed by Knittelfelder et al. [27].

On the other hand, the absence of chromatography can be partially overcome by the application of an ion mobility interface [47,48]. The applied setup is fully compatible with mounting of such a module, which can provide significant spectrum cleaning due to the gas-phase preseparation of ionized analyte molecules, thereby improving sensitivity and identification confidence.

The application of one-phase lipid extraction with an alcohol-enriched extraction solvent represents a further advantage of the developed workflow, which enables the extraction and detection/quantification of more polar molecules, such as lower molecular weight organic acids, sugars, or lipid metabolic turnover products. Quantitative assessment of these compounds by the novel platform is in progress in our laboratory. Moreover, the extraction solvent also enables the detection of higher brain gangliosides, such as disialo- (GD) and trisialo (GT) species (Figure S3C). These highly polar acidic glycosphingolipids partition into the aqueous phase and are therefore often discarded upon the traditional two-phase lipid extractions or can be recovered from the aqueous phase via a time-consuming process [49].

Recently, Mund et al. demonstrated the performance of deep visual proteomics, which combines artificial intelligence (AI)-based single-cell phenotyping with automated LMD and ultra-high-sensitivity proteomics [25]. In this platform, AI-driven single-cell phenotyping is based on the Biology Image Analysis Software ((BIAS) Single Cell Technologies, Szeged, Hungary), which can be readily combined with the described LMD-coupled shotgun lipidomic method. The program is capable to coordinate the scanning and LMD microscopes and allows single-cell classification using machine learning-based algorithms. Preliminary data suggest that the integration of this AI-driven image analysis tool into the current workflow can significantly extend its applicability.

## 4. Conclusions

The novel LMD-coupled shotgun lipidomic platform we present here offers quantitative and reliable analysis of the lipidome with broad-range lipid species coverage from thin tissue cryosections down to 5000 µm^2^ area in size. Although this spatial resolution is far behind the resolution power of MSI, it can be well-suited for a wide range of applications from cell culture models to tissue cryosections as demonstrated in this work. In addition, further successive cryosections can be subjected to other omics and/or staining procedures, thereby establishing a multiomics platform. The integration of lipidomic results with transcriptomic, proteomic, and immunohistological data can improve our understanding of tissue heterogeneity as well as of the pathology of various lipid-related disorders.

## Data Availability

All data generated or analyzed during this study are included in this published article and its Supplementary Materials.

## Supplementary Materials

The following supporting information can be downloaded at: https://www.mdpi.com/article/10.3390/cells12030428/s1. Figure S1: Representative MS1 survey spectra acquired with polarity switching from a liver LMD spot; Figure S2: Comparison of the species profile of the protonated ([M+H]+) vs. chloride adduct ([M+Cl]−) forms of PC acquired with polarity switching from liver full cryosections; Figure S3: (A) MS acquisition method setup with polarity switching extended with segments for lower mass ranges and fragmentations. (B) Examples for MS2 and MS3 fragmentation experiments to determine the fatty acyl composition of glycerophospholipids from a liver LMD spot. (C) Detection of disialo- (GD) and trisialo- (GT) ganglioside species from a hippocampal LMD area as doubly and triply charged ions, respectively; Table S1: Mass spectrometry quantification details; Table S2: Compositional data for the mouse liver lipidome (mol% of polar lipids); Table S3: Absolute lipid content data for mouse kidney samples; Table S4: Compositional data for the mouse kidney lipidome; Table S5: Absolute lipid content data for HeLa 2D cell cultures; Table S6: Compositional data for HeLa 2D cell culture lipidome; Table S7: Compositional data for the mouse hippocampus lipidome.
