# Supplementary material for: Development of a Laser Microdissection-Coupled Quantitative Shotgun Lipidomic Method to Uncover Spatial Heterogeneity
Source: Cells. 2023 Jan 28;12(3):428. doi: 10.3390/cells12030428 (PMC9913738; doi:10.3390/cells12030428)
Supplement: Supplementary file 1 [file cells-12-00428-s001.zip › Zsiros_et_al_SupplFigs_2022.pdf]

cells

Article

Development of a Laser Microdissection-Coupled Quantitative Shotgun Lipidomic Method to Uncover Spatial Heterogeneity

Vanda Varga-Zsíros [1,2], Ede Migh [1], Annamária Marton [1], Zoltán Kóta [3], Csaba Vizler [1], László Tiszlavicz [4], Péter Horváth [1], Zsolt Török [1], László Vígh [1], Gábor Balogh [1,*,†] and Mária Péter [1,†]

1 Biological Research Centre, Institute of Biochemistry, Eötvös Loránd Research Network, 6726 Szeged, Hungary
2 University of Szeged, Faculty of Science and Informatics, Ph.D. School in Biology, 6726 Szeged, Hungary
3 Hungarian Centre of Excellence for Molecular Medicine, Single Cell Omics Advanced Core Facility, 6726 Szeged, Hungary
4 University of Szeged, Albert Szent-Györgyi Medical Centre, Department of Pathology, 6725 Szeged, Hungary
* Correspondence: balogh.gabor@brc.hu
† These authors contributed equally.

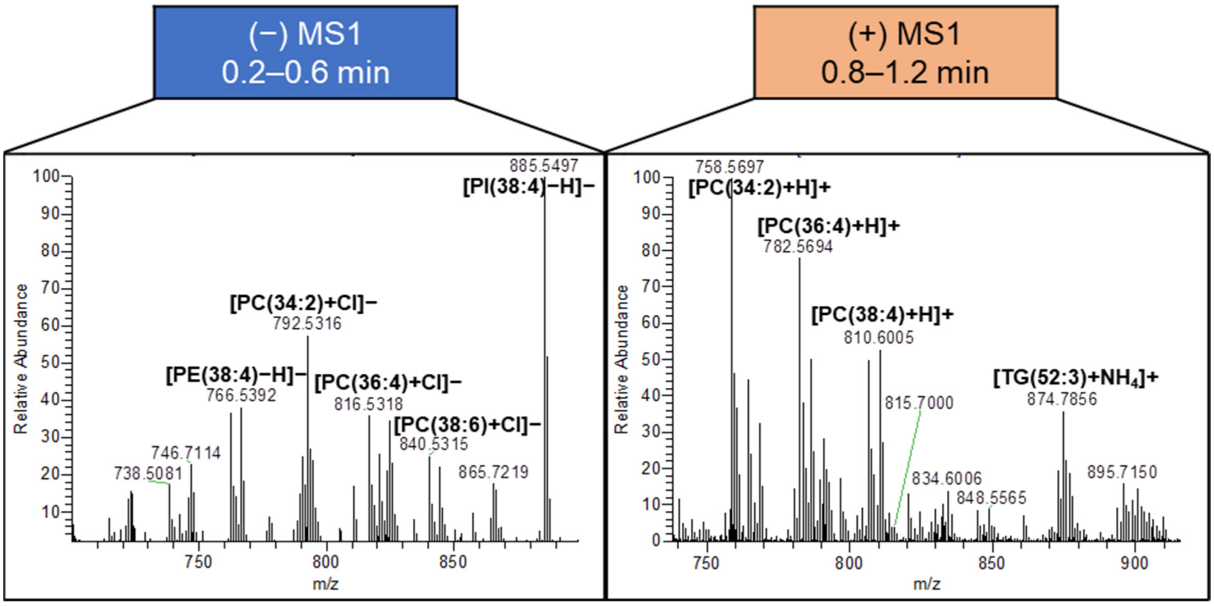

Figure S1. Representative MS1 survey spectra acquired with polarity switching from a liver LMD spot.

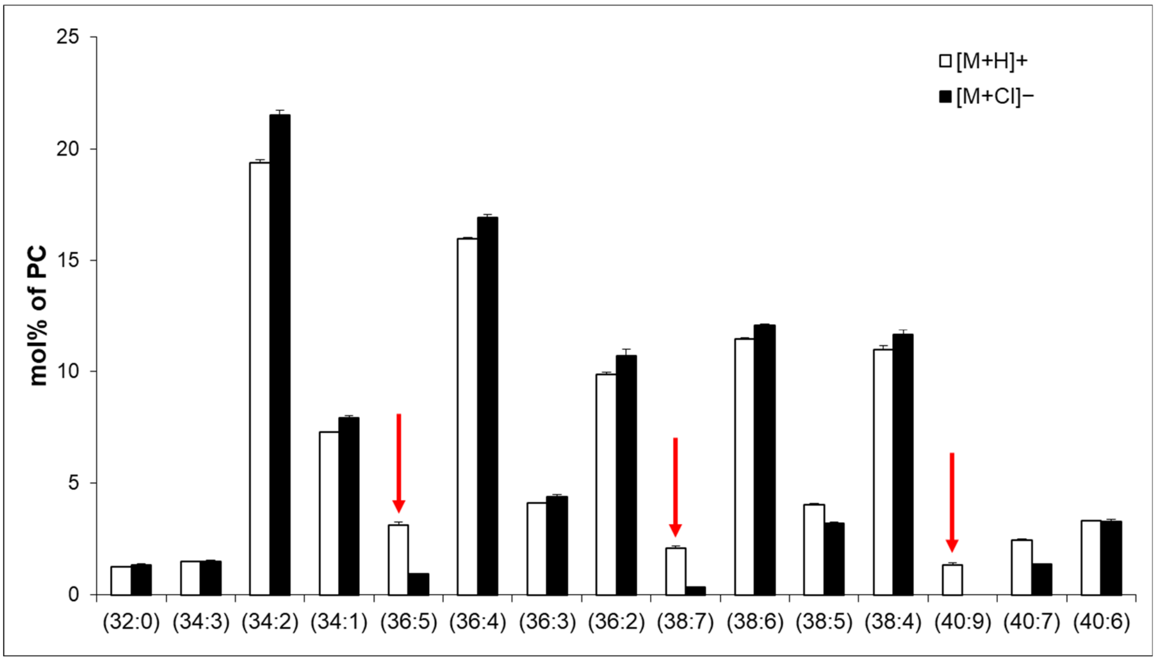

Figure S2. Comparison of the species profile of the protonated ([M+H]+) vs. chloride adduct ([M+Cl]−) forms of PC acquired with polarity switching from liver full cryosections. Red arrows show the overestimation of certain polyenoic species (e.g., PC(36:5), PC(38:7), or PC(40:9)) from the positive polarity mode due to isobaric overlap with sodiated ions. Data represent mean ± SD (n = 4). PC, phosphatidylcholine.

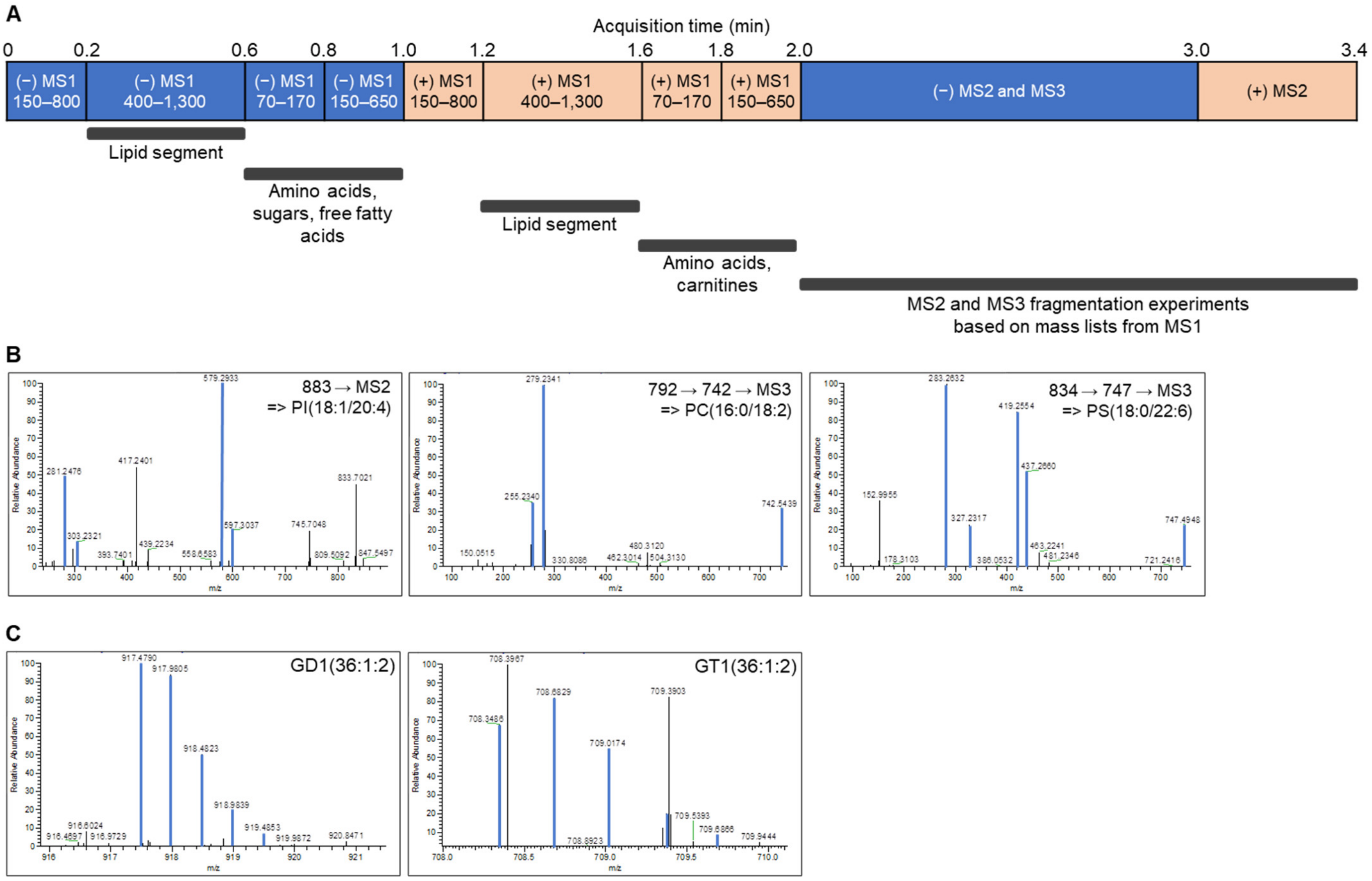

Figure S3. (A) MS acquisition method setup with polarity switching extended with segments for lower mass ranges and fragmentations. (B) Examples for MS2 and MS3 fragmentation experiments to determine the fatty acyl composition of glycerophospholipids from a liver LMD spot. Left, MS2 for 883.53 ([PI(38:5)−H]−) with CID35, which produces 281.25 (loss of 18:1 carboxylate anion), 303.23 (loss of 20:4 carboxylate anion), 579.29 (loss of neutral 20:4 fatty acid), and 597.30 (loss of neutral 20:4 ketene) fragment ions leading to the resolved formula of PI(18:1/20:4). Center, MS3 for 792.53 ([PC(34:2)+Cl]) with CID30, which produces 742.54 due to the neutral loss of MeCl followed by HCD25, which generates 255.23 (loss of 16:0 carboxylate anion) and 279.23 (loss of 18:2 carboxylate anion) fragments leading to the resolved formula of PC(16:0/18:2). Right, MS3 for 834.53 ([PS(40:6)−H]−) with CID30, which produces 747.50 due to the neutral loss of a 87-Da characteristic fragment followed by HCD25, which generates 283.26 (loss of 18:0 carboxylate anion), 327.23 (loss of 22:6 carboxylate anion), 419.26 (loss of neutral 22:6 fatty acid), and 437.27 (loss of neutral 22:6 ketene) fragment ions leading to the resolved formula of PS(18:0/22:6). (C) Detection of disialo- (GD) and trisialo- (GT) ganglioside species from a hippocampal LMD area as doubly and triply charged ions, respectively.
